# Stress-induced inactivation of the *Staphylococcus aureus* purine biosynthesis repressor leads to hypervirulence

**DOI:** 10.1038/s41467-019-08724-x

**Published:** 2019-02-15

**Authors:** Mariya I. Goncheva, Ronald S. Flannagan, Brigid E. Sterling, Holly A. Laakso, Nancy C. Friedrich, Julienne C. Kaiser, David W. Watson, Christy H. Wilson, Jessica R. Sheldon, Martin J. McGavin, Patti K. Kiser, David E. Heinrichs

**Affiliations:** 1 Department of Microbiology and Immunology, University of Western Ontario, London, ON Canada N6A 5C1; 2 Department of Pathology, University of Western Ontario, London, ON Canada N6A 5C1; 3 Present Address: Department of Pathology, Microbiology, and Immunology, Vanderbilt University Medical Center, Nashville, TN USA

## Abstract

*Staphylococcus aureus* is a significant cause of human infection. Here, we demonstrate that mutations in the transcriptional repressor of purine biosynthesis, *purR*, enhance the pathogenic potential of *S. aureus*. Indeed, systemic infection with *purR* mutants causes accelerated mortality in mice, which is due to aberrant up-regulation of fibronectin binding proteins (FnBPs). Remarkably, *purR* mutations can arise upon exposure of *S. aureus* to stress, such as an intact immune system. In humans, naturally occurring anti-FnBP antibodies exist that, while not protective against recurrent *S. aureus* infection, ostensibly protect against hypervirulent *S. aureus* infections. Vaccination studies support this notion, where anti-Fnb antibodies in mice protect against *purR* hypervirulence. These findings provide a novel link between purine metabolism and virulence in *S. aureus*.

## Abstract (web summary)

PurR acts as transcriptional repressor of purine biosynthesis genes in some bacterial species. Here, the authors show that *purR* mutations can arise in *Staphylococcus aureus* upon exposure to stress, leading to upregulation of fibronectin-binding proteins and increased virulence.

## Introduction

In humans, *Staphylococcus aureus* may exist as a commensal bacterium or as a pathogen. Data from the United States Centers for Disease Control and Prevention show that approximately one-third of the US population is colonized with *S. aureus*^1^, and colonization with *S. aureus* is associated with increased risk of subsequent infection^2^. Infections caused by *S. aureus* range in severity from relatively minor skin and soft tissue infections through to invasive diseases such as pneumonia, infective endocarditis and osteomyelitis^3^. Strikingly, the magnitude of morbidity and mortality caused by *S. aureus* is highlighted by reports that, in the US, invasive infections by this bacterium cause more deaths than HIV^4^.

That *S. aureus* can infect virtually any organ or tissue in the body is a reflection of its vast repertoire of virulence factors that contribute to bacterial pathogenesis through mechanisms involving tissue adherence^5,6^, cellular intoxication^7–9^, and immune modulation and deception^10,11^. Virulence factor expression in *S. aureus* is complex and coordinately regulated by multiple transcription factors, regulatory RNAs, two-component sensing systems and quorum-sensing^12–14^. Despite a wealth of knowledge on virulence regulation in *S. aureus*, there are still outstanding questions to be resolved, as novel mechanisms of virulence regulation are still being discovered, especially with regard to environmental or metabolic cues to which *S. aureus* responds^15^.

Exposure to elevated temperatures, for example 42 °C, a temperature frequently used to cure *S. aureus* of recombinant plasmids during mutagenesis procedures, can select for mutations in the *S. aureus* genome. Mutations in the global two-component regulator SaeRS have previously been isolated following mutagenesis^16^, and mutations in the *sae* regulatory system show drastically reduced toxin production and have attenuated virulence^17–20^. Screening for unintended *sae* mutations is straight forward, as the mutants are easily identified as having reduced haemolytic activity on blood agar plates. Little is known, however, about other unintended secondary mutations that may be selected for in response to stress, especially those that may impact on the virulence potential of *S. aureus*.

In this study, we identify mutations that occur in the *S. aureus purR* gene in response to stress, including growth at elevated temperatures (i.e. 42 °C). The function of *purR* in *S. aureus* has not been characterized, but the gene is homologous to those that encode the purine biosynthesis repressors in *Bacillus subtilis* and *Escherichia coli*; we show here that mutations in *purR* do indeed result in upregulation of purine biosynthetic genes in *S. aureus*. However, we further demonstrate that *purR* mutations have a heretofore undescribed role in regulating expression of *S. aureus* fibronectin binding proteins and, thus, play a role in the interaction of *S. aureus* with fibronectin. In the absence of anti-FnbA/B antibodies, *purR* mutants clump vigorously in serum in a fibronectin-dependent manner. Importantly, *purR* mutants, also through FnbA/B-dependent mechanisms, are hypervirulent in a systemic model of infection in mice, and vaccination of mice against FnbA/B can diminish hypervirulence and ameliorate animal mortality. We suggest that, in *S. aureus*, PurR is critical to limit the expression of *fnb* genes, known to be maximally expressed at low cell density, in order to promote colonization, yet prevent cell clumping in the vasculature.

## Results

### *S. aureus purR* mutants vigorously clump during growth in serum

**Fig. 1:**
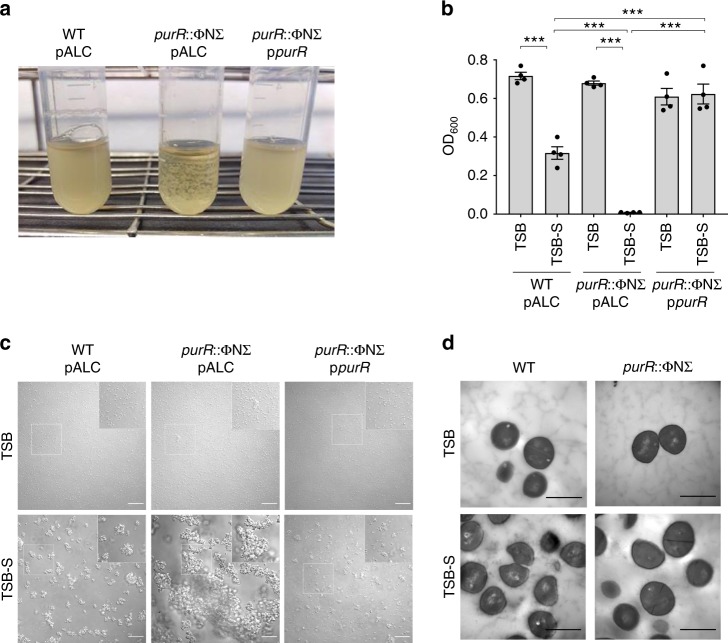
Disruption of *purR* causes cell clumping of *S. aureus* USA300. In **a**, representative images of USA300, USA300 *purR*::ΦNΣ or the complemented *purR*::ΦNΣ mutant in culture tubes following growth in TSB with 10% (v/v) horse serum (TSB-S) for 3.5 h from a starting OD_600_ equivalent of 0.03. In **b**, graphical representation of the relative sedimentation of bacterial aggregates in cultures as grown in **a**, reflected by the OD_600_ values of the centre of liquid cultures after sitting without shaking for 5 min following shaking at 37 °C for 3.5 h. Data are mean ± SEM of four independent experiments. ***p-value < 0.001, based on a one-way analysis of variance (ANOVA) with a Bonferroni post-test. In **c**, the representative micrographs show bacterial cell clusters that arise during growth in TSB or TSB-S. White boxes define the region of interest that is depicted in the insets. Scale bars  = 40 µm. In **d**, transmission electron micrographs are shown for *S. aureus* USA300 and the USA300 *purR*::ΦNΣ strain grown in the presence (TSB-S) or absence (TSB) of horse serum. The representative images depict cells at ×11,000 magnification. Scale bars  = 1 µm. Source data are provided as a Source Data file

In our laboratory, we generate deletion mutations in iron-regulated genes and test mutants for growth in chemically defined media (e.g. RPMI-1640) containing 10% v/v horse serum (HS) to induce iron starvation. Over time, we noted that a number of mutants, in the USA300 genetic background, would clump vigorously when grown in the presence of HS, a trait not observed for WT USA300. The hallmark of this phenotype was that, during growth, visibly large clumps would appear in the culture and, when the culture tube was allowed to sit without shaking, the clumped material would settle to the bottom of the tube within minutes. This response was independent of iron starvation as robust clumping occurred when the bacteria were grown in tryptic soy broth, an iron replete medium, containing 10% v/v HS (TSB-S). To investigate this phenotype further, we performed whole genome sequencing on one of these clumping mutants and identified a non-synonymous single nucleotide polymorphism (SNP) in the *purR* gene causing a Q52P mutation (*purR*^Q52P^). The *purR* gene is homologous to those encoding the purine biosynthesis repressors in *E. coli* and *B. subtilis* but, to date, has not been studied in *S. aureus*. We independently discovered a second clumping mutant while generating a completely separate markerless deletion in the USA300 genome. We PCR-amplified the *purR* gene and discovered it carried a deletion of a guanine at position 682 of the gene, causing a frameshift in the protein after V229. To confirm that loss of *purR* indeed correlated with the hyper-clumping phenotype, we mobilized the *purR*::ΦNΣ mutation from the Nebraska transposon mutant library^21^ into our laboratory USA300 strain (hereafter referred to as *purR*::ΦNΣ). The *purR*::ΦNΣ strain demonstrated similar clumping to the SNP-containing strain and the phenotype could be fully complemented by providing *purR in trans* on a multi-copy plasmid (referred to as p*purR*) (Fig. 1a). Given that cultures containing clumped bacteria, when allowed to sit without shaking, rapidly clarify due to sedimentation of the cells in culture tubes, we developed an assay to quantitate relative clumping by measuring the culture optical density (see the Methods section). This analysis detected a significant decrease in OD_600_ values for both WT USA300 and *purR*::ΦNΣ in TSB-S, when compared to TSB alone. However, bacterial sedimentation (i.e. clumping) was greatly enhanced for *purR*::ΦNΣ in serum as compared to WT USA300 (Fig. 1b). Furthermore, these measurements confirmed that provision of *purR in trans* completely reversed the clumping phenotype (Fig. 1b).

To study the hyper-clumping phenotype further, we used brightfield microscopy to examine the cells grown in TSB or TSB-S. WT USA300 and the complemented *purR*::ΦNΣ mutant grown in TSB formed only small ‘grape-like’ clusters of 2–4 cells, as expected for *S. aureus*. In contrast, *purR*::ΦNΣ formed aggregates comprised of greater numbers of cells, including some noticeably larger clusters that were not observed for WT bacteria (Fig. 1c, top panels). Consistent with what is known of the interaction of *S. aureus* with serum proteins^22,23^, USA300 and the *purR*::ΦNΣ complemented strains, grown in TSB-S as compared to TSB alone, formed larger cell clusters due to aggregation of the bacteria through binding of serum proteins (Fig. 1c, bottom panels). In contrast, bacterial aggregation was greatly exaggerated for *purR*::ΦNΣ, where aggregated masses of bacteria took up the majority of the field of view (Fig. 1c, bottom panel), undoubtedly related to the macroscopic sedimentation observed in liquid cultures.

To assess whether cell clumping could be caused by cell division defects in the *purR*::ΦNΣ background, we performed transmission electron microscopy of WT or *purR*::ΦNΣ mutant cells grown in TSB or TSB-S. For both strains, irrespective of culture conditions, division septa were visible and the apparent cell morphology did not differ, indicating cell division defects were not present in *purR*::ΦNΣ (Fig. 1d). We therefore next hypothesized that the robust aggregation of *purR* bacteria was mediated by specific bacterial factors. Interestingly, no discernible differences in protein profiles or growth were observed between WT and *purR*::ΦNΣ bacteria at various growth phases (Supplementary Figure 1a, b, c). RNAseq on mid-exponential (OD_600_ = 1.0) phase cultures grown in TSB showed that genes of the purine biosynthesis pathway were elevated in the *purR*::ΦNΣ strain, as compared to the WT, however, few other differences could be detected between the two genotypes (Supplementary Table 1). RT-PCR was performed on a select number of genes and the data agreed with the RNAseq data, where *purE*, the first gene in the *purEKCSQLFMNHD* purine biosynthetic operon, demonstrated the greatest transcriptional increase (Supplementary Figure 1d). Altogether, these data demonstrate that *purR* regulates the purine biosynthesis pathway of *S. aureus* and inactivation of *purR* leads to exaggerated serum-dependent cell clustering. However, these analyses failed to identify an obvious effector responsible for the clumping phenotype.

### Serum clumping requires fibronectin binding proteins

We next analysed whether the *purR* phenotype was conserved across different *S. aureus* backgrounds. To this end, we transduced the *purR*::ΦNΣ mutation into *S. aureus* strains RN6390, SH1000, MN8 and Newman and complemented each mutant. Similar to USA300, growth of the RN6390, SH1000 and MN8 *purR* mutants in TSB-S demonstrated vigorous cell clumping and, for each strain, provision of *purR in trans* complemented the phenotype (Supplementary Figure 2a, b, c). In contrast, Newman *purR*::ΦNΣ failed to hyper-aggregate in the presence of HS and was indistinguishable from WT Newman when grown in either TSB or TSB-S (Supplementary Figure 2d). Of note, strain Newman expresses mutated fibronectin binding proteins (FnBPs; FnbA and FnbB) that, unlike in other *S. aureus* strains, are not cell wall anchored^24^, leading us to hypothesize that cell wall anchored FnbA/B may be required for hyper-clumping.

**Fig. 2:**
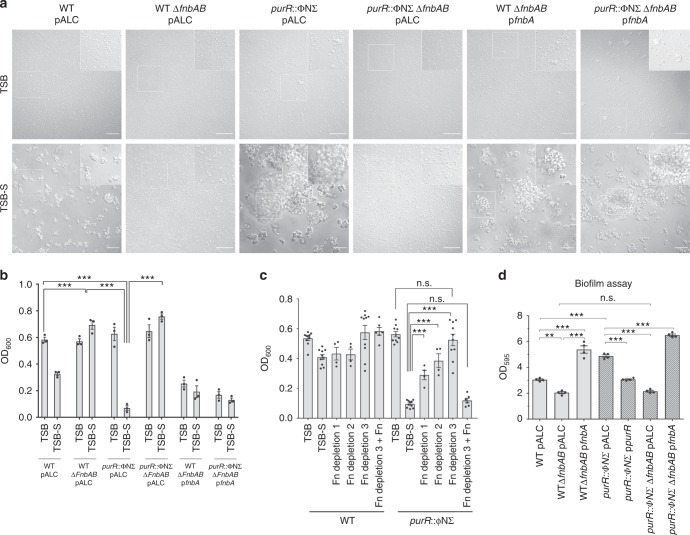
The *purR*-dependent clumping phenotype requires fibronectin binding proteins and host fibronectin. In **a**, cultures were grown in TSB or TSB-S for 3.5 h and then imaged on a wide field microscope at ×40 magnification. White boxes define the region of interest that is depicted in the insets. Scale bars = 40 µm. Representative images are shown. In **b**, cultures were grown as in **a** and OD_600_ was measured as described in the legend to Fig. 1 and in the Methods section. Data shown are mean ± SEM of four independent experiments. ***p-value < 0.001, based on a one-way ANOVA with a Bonferroni post-test. In **c**, WT and the *purR*::ΦNΣ mutant were grown in TSB, TSB-S, TSB containing 10% v/v of various levels of Fn-depleted horse serum or Fn-depleted horse serum with the addition of eluted fibronectin (Fn depletion 3+Fn). Measurement of OD_600_ of cultures to evaluate clumping was performed as described above. Data shown are mean ± SEM of five independent experiments and two different Fn purifications. ***p-value < 0.001, based on a one-way ANOVA with a Bonferroni post-test. In **d**, biofilm forming ability of indicated strains was measured after growth in TSB in a standard 96-well plate biofilm assay (see the Methods section). Data shown are mean ± SEM of four experiments. **p-value < 0.01, ***p-value < 0.001, based on a one-way ANOVA with a Bonferroni post-test. Source data are provided as a Source Data file

To directly test the involvement of the FnBPs in *purR*-dependent clumping, we engineered, in WT and *purR*::ΦNΣ USA300 bacteria, markerless deletions of the tandemly-duplicated *fnbA* and *fnbB* genes. Growth of the resulting *purR*::ΦNΣ *fnbA/B* mutants in TSB and TSB-S did not differ from that of WT USA300 and, notably, serum-dependent hyper-clumping did not occur (Fig. 2a, b). Indeed, the USA300Δ*fnbAB* construct exhibited less serum-dependent clumping than WT, demonstrating the importance of these proteins in normal interactions of *S. aureus* with serum components (Fig. 2a). Of note, complementation of the Δ*fnbAB* mutants with *fnbA* on an overexpression plasmid resulted in exaggerated clumping during growth in TSB without serum (Fig. 2a, b), likely due to the increased number of homophilic interactions between FnbA molecules, which have previously been reported to contribute to bacterial aggregation^25^. Overall, these data show that hyper-clumping due to *purR* inactivation occurs in several *S. aureus* strains and requires cell wall-anchoring of FnBPs.

### Serum clumping by *purR* mutants requires fibronectin

The multifunctional *S. aureus* FnBPs bind to fibrinogen, fibronectin and elastin^5^. To determine which serum component was involved in the clumping phenotype, we allowed *purR*::ΦNΣ bacteria to grow in TSB-S and form clumps. We isolated the clumped material and used mass spectrometry to identify enriched serum proteins that copurified with the bacteria (see the Methods section). These analyses revealed only one protein, fibronectin (Fn) from *Equus ferus przewalskii* (Mongolian wild horse) was significantly enriched in *purR*::ΦNΣ derived samples. To confirm the involvement of Fn in *purR*-dependent hyper-clumping, soluble Fn was removed from horse serum by serial passage over a gelatin sepharose column (Supplementary Figure 3). When the Fn-depleted serum was used in clumping assays, we observed a decrease in the hyper-clumping phenotype of the *purR*::ΦNΣ mutant (Fig. 2c). Reconstitution of the Fn-depleted serum with the purified horse Fn restored *purR*-dependent clumping to normal levels (Fig. 2c). Together these data demonstrate the *purR-*dependent clumping in serum requires *S. aureus* FnBPs and host Fn.

### *S. aureus purR* mutants demonstrate enhanced biofilm formation

The clustering of *purR*::ΦNΣ cells in TSB, coupled with the dependency of the aggregation phenotype on FnBPs, lead us to hypothesise that *purR*::ΦNΣ bacteria were better able to initiate biofilm formation. In agreement, *purR*::ΦNΣ bacteria formed increased biofilm as compared to WT USA300 (Fig. 2d), and this phenotype could be eliminated by the deletion of *fnbAB* in the *purR*::ΦNΣ background (Fig. 2d). Moreover, deletion of *fnbAB* genes eliminated any differences between WT and *purR*::ΦNΣ cells and diminished biofilm formation. Conversely, overexpression of *fnbA* from a plasmid enhanced biofilm formation, irrespective of *purR*.

### PurR represses transcription of the *purE* operon and *fnbAB*

**Fig. 3:**
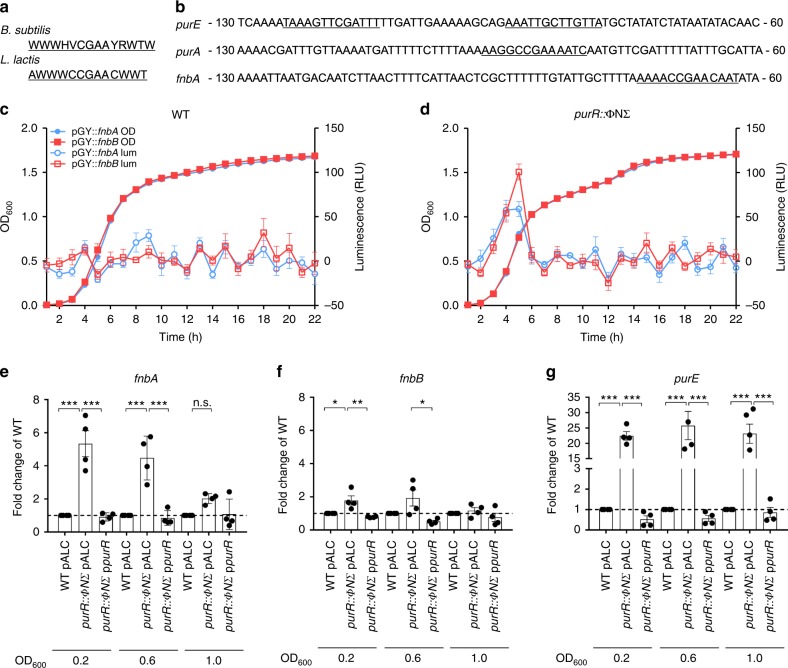
*purR* mutations lead to transcriptional upregulation of the purine biosynthesis operon and *fnbAB*. **a** Consensus PurBox sequence for *B. subtilis* and *L. lactis* (data adapted from refs. ^26,27^). **b** Promoter sequences of *purE*, *purA* and *fnbA*, with putative PurBox sequences underlined. WT (**c**) or *purR*::ΦNΣ mutant (**d**) containing a luciferase construct with the promoter sequence of *fnbA* or *fnbB* (see the Methods section) were grown in TSB and OD_600_ and luminescence monitored. Data shown are mean ± SEM of three experiments. In **e**, **f** and **g**, indicated strains were grown to OD_600_ of 0.2, 0.6 or 1.0, total RNA was extracted and RT-PCR analysis performed for relative abundance of *fnbA* (**e**), *fnbB* (**f**) and *purE* (**g**) transcripts. All data were normalized to levels of *rpoB* and expressed as fold change using WT pALC (empty plasmid) as comparator at each OD_600_ value. Data shown are mean ± SEM of four independent experiments. *p-value < 0.05, **p-value < 0.01, ***p-value < 0.001, based on a one-way ANOVA with a Bonferroni post-test. Source data are provided as a Source Data file

How inactivation of *purR* is connected to FnBP function and/or expression was not understood, since our RNAseq analysis failed to detect changes in either *fnbA* or *fnbB* transcript levels at culture densities of OD_600_ of 1.0. Studies in *B. subtilis* and *Lactococcus lactis* have identified conserved sequence motifs in promoter regions, named PurBoxes, where PurR binds. Single or double PurBoxes can be present, and double PurBoxes are often palindromic, but all contain a central conserved CGAA motif^26,27^ (Fig. 3a). Analysis of the *S. aureus* genome identified sequences similar to those in *B. subtilis* and *L. lactis* upstream of the *purE* and *purA* genes in *S. aureus* USA300 (Fig. 3b) and, not surprisingly, these genes are upregulated in the *purR*::ΦNΣ strain (see Supplementary Table 1). Remarkably, a similar putative PurR-binding sequence was also present upstream the *fnbA* and *fnbB* genes (Fig. 3b). To determine whether transcription of *fnbA* and *fnbB* is influenced by PurR we generated plasmids carrying the *fnbA* and *fnbB* promoters fused to a promoterless *lux*-gene construct and monitored bioluminescence in WT and *purR*::ΦNΣ bacteria. Bioluminescence could not be detected above background levels in WT cells, presumably due to low levels of transcription from the *fnb*A/B promoters (Fig. 3c). In contrast, bioluminescence was detected for both the *fnbA* and *fnbB* promoter constructs in the *purR*::ΦNΣ mutant, where luminescence peaked at a culture density of OD_600_ 0.5–0.6 (Fig. 3d). Accordingly, we investigated transcript levels of *fnbA* and *fnbB* at early growth phases. Relative to WT, *fnbA* transcripts were elevated in the *purR*::ΦNΣ mutant, at culture densities as low as OD_600_ of 0.2 (Fig. 3e), and steadily decreased as the culture density increased. Consistent with our RNAseq analysis, no significant differences in *fnbA* transcripts were present between the WT and the *purR*::ΦNΣ mutant at an OD_600_ of 1.0. Of note, *fnbB* transcripts were only elevated in the *purR*::ΦNΣ mutant at OD_600_ of 0.2 (Fig. 3f). Consistent with de-repression due to the absence of its regulator/repressor, and concordant with our previous data, *purE* transcripts were upregulated at all time points tested (Fig. 3g).

### *S. aureus purR* mutants are hypervirulent

**Fig. 4:**
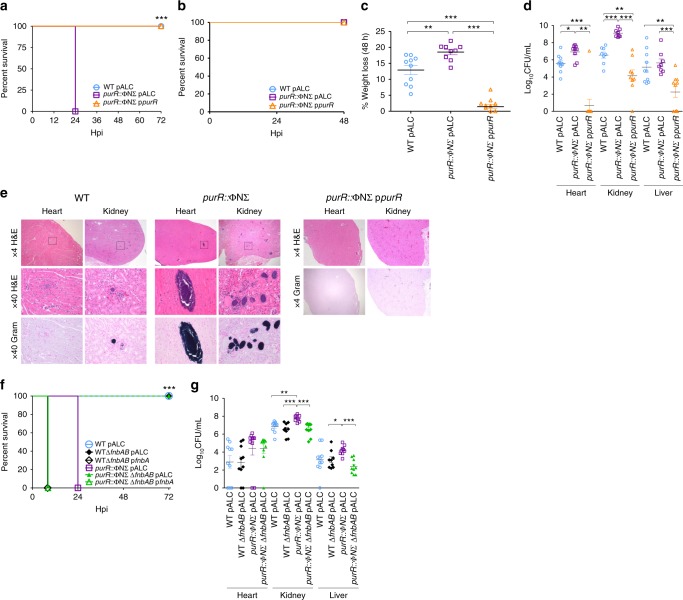
A *S. aureus purR* mutant is hypervirulent via FnbAB. In **a**, mice (9–12 per group) were infected with ~1 × 10^7^ CFU of WT USA300, USA300 *purR*::ΦNΣ or complemented *purR*::ΦNΣ mutant and survival monitored over 72 h. ***p-value < 0.001, based on a Mantel-Cox test. In **b**, animals were infected as in **a**, but with 2–2.5 × 10^6^ CFU, and **c** weight loss monitored daily for 48 h. **p-value < 0.01, ***p-value < 0.001, based on a one-way ANOVA with a Bonferroni post-test. In **d**, animals from **b** were sacrificed at 48 h post infection (hpi), and heart, kidney and liver were harvested and bacterial burdens determined. Data shown are mean ± SEM, *p-value < 0.05, **p-value < 0.01, ***p-value < 0.001, based on a Student’s unpaired *t*-test. In **e**, two animals per bacterial strain were infected as in **a**, with ~1 × 10^7^ CFU, sacrificed at 24 hpi and organs harvested. Organs were paraffin embedded, sectioned and stained with H&E and a Gram stain. Representative images are shown. In **f**, animals were infected as in **a**, with ~1 × 10^7^ CFU, with the inclusion of WTΔ*fnbAB* and *purR*::ΦNΣΔ*fnbAB* strains, and monitored for 72 h. ***p-value < 0.001, based on a Mantel-Cox test. In **g**, the heart, kidney and liver from the animals infected in **e** were harvested at the point of sacrifice and bacterial burden determined. Data shown are mean ± SEM, *p-value < 0.05, **p-value < 0.01, ***p-value < 0.001, based on a Student’s unpaired *t*-test. Source data are provided as a Source Data file

Given the strong Fn-binding phenotype associated with a *S. aureus purR* mutant, we evaluated the virulence potential of the mutant. Mice were infected via the tail vein with WT USA300, the *purR*::ΦNΣ mutant, and the *purR*::ΦNΣ mutant complemented with p*purR* at a dose of ~1 × 10^7^ CFU. Remarkably, 100% of the mice infected with the *purR*::ΦNΣ mutant met humane endpoint criteria by 24 hpi, whereas 100% of the mice infected with either the WT or complemented mutant survived past 72 hpi (Fig. 4a). The *purR*^*Q52P*^ mutant demonstrated the same hypervirulent phenotype as the *purR*::ΦNΣ mutant (Supplementary Figure 4a).

In subsequent experiments, we tested the effect of a lower dose of the *purR*::ΦNΣ mutant and found that infection with ~2 × 10^6^ CFU allowed survival up to 48 hpi (Fig. 4b). At 48 hpi, we observed significantly greater weight loss and increased bacterial burdens in mice infected with the *purR*::ΦNΣ mutant when compared to those infected with WT (Fig. 4c, d). The mice infected with the complemented strain showed statistically significant decreases in weight loss and bacterial burden, even compared to mice infected with WT.

Histopathological analysis of animals infected with a high dose of WT (~1 × 10^7^ CFU) for 24 h demonstrated lesions predominantly in the heart and the kidney (Fig. 4e). Animals infected with the *purR*::ΦNΣ mutant had larger and more frequent lesions in both the heart and kidneys (Supplementary Table 2), with multifocal necrotic areas, often centred on discrete groups of Gram-positive bacteria (Fig. 4e). Complementation of the *purR*::ΦNΣ mutant almost completely eliminated the formation of lesions (Fig. 4e), concurring with the decreased bacterial burden previously observed.

To confirm the role of *fnbAB* in the *purR* hypervirulence phenotype, we infected mice with the Δ*fnbAB* mutant, in either the WT or *purR*::ΦNΣ background. While *purR*::ΦNΣ infected animals required sacrifice by 24 hpi the deletion of *fnbAB* in that background completely ablated the hypervirulent phenotype (Fig. 4f). Of note, infections with strains carrying an *fnbA* overexpression plasmid, indifferent of the *purR* background, resulted in very rapid effects on animal health, and animals required euthanasia by ~6–8 hpi (Fig. 4f). This demonstrates the profound effects of aberrant *fnb* overexpression, suggesting that even transient upregulation of FnBPs has a severe impact on disease severity in a systemic infection model. Bacterial burdens in the hearts, kidneys and livers of the remaining groups were in agreement with the survival data, with increased numbers of bacteria for the *purR*::ΦNΣ strain, but not for the *purR*::ΦNΣ Δ*fnbAB* strain, compared to WT (Fig. 4g) (CFU for p*fnbA* carrying strains were not determined). Of note, no difference in survival or bacterial burden was observed between the WT and Δ*fnbAB* strains, indicating that while these proteins are not required for pathogenesis of WT USA300 in a systemic model of infection, they are indispensable for the hypervirulent phenotype of the *purR*::ΦNΣ mutant. In agreement with the requirement of FnbAB for *purR*-dependent hypervirulence, a *purR*::ΦNΣ mutant in strain Newman was not hypervirulent in this model (Supplementary Figure 4b).

### Mutations in *purR* occur at elevated temperatures and in vivo

The *purR*^Q52P^ SNP and the *purR*^V229frameshift^ SNP-containing strains were isolated following allelic replacement mutagenesis techniques, a process that has previously been reported to select for mutations in the virulence regulatory genes *saeRS*^16^. Plasmids for allelic replacement are often temperature sensitive and curing of plasmids following homologous recombination necessitates growth at elevated temperatures. To investigate if exposure to high temperatures also selects for *purR* mutations in *S. aureus*, we constructed a reporter strain that colorimetrically identifies *purR* mutants. Given that the promoter of the purine biosynthetic operon (*purEKCSQLFMNHD*) was highly expressed in a *purR* mutant, we fused the promoter of this operon to a promoterless *gusA* gene, encoding ß-glucuronidase (referred to as P_*purE::gusA*_) (Supplementary Figure 5a), and inserted this fusion into the genome (see the Methods section). When cultured on X-Gluc-containing solid media, USA300 P_*purE::gusA*_ colonies were pale yellow while the USA300 *purR*::ΦNΣ strain carrying the genomic reporter were dark blue; this indicates that the reporter was capable of identifying *purR* mutants in culture. To define whether we could identify naturally-occurring *purR* mutants, we cultured USA300 P_*purE::gusA*_ at either 37 °C or 42 °C, with daily passage, for 5 days. Blue colonies were only detected from cultures grown at 42 °C (Supplementary Figure 5b), at a frequency of ~0.1–1.0% following 5 days of passaging (Supplementary Figure 5b). Sequencing of the *purR* gene from select blue colonies identified a variety of additional mutations in *purR* (Y71Stop, V156E, S172Stop, H225D, S230Stop, Q240Stop).

The in vivo environment also presents a strong selection pressure on bacteria. Therefore, we were interested to determine if passage of *S. aureus* through mice would select for *purR* mutants. Unfortunately, for unknown reasons, the USA300 P_*purE::gusA*_ construct was lost from the genome without antibiotic selection in vivo. Therefore, we tested colonies recovered from the organs of mice infected with WT USA300 for 4 days for clumping in TSB-S (in a 96-well plate format). Potential mutants were phenotypically confirmed in a tube assay and the *purR* gene sequenced. A mutant with a R96A SNP was identified from an infected kidney, and demonstrated cell clustering in TSB (Supplementary Figure 5c) and clumping in TSB-S (Supplementary Figure 5d). The phenotype could be complemented by the introduction of p*purR*, indicating the SNP was solely responsible for the observed phenotype (Supplementary Figure 5c, d). It was of interest whether strains containing SNPs in *purR*, and recovered from murine infections, also displayed the characteristic hypervirulence we described here. Infection of mice with the *purR*^R96A^ SNP resulted in the same hypervirulent phenotype as the *purR*::ΦNΣ mutant (Supplementary Figure 5e), with animals requiring sacrifice within 24 hpi.

### Anti-FnBP antibodies ameliorate *purR* mutant clumping

**Fig. 5:**
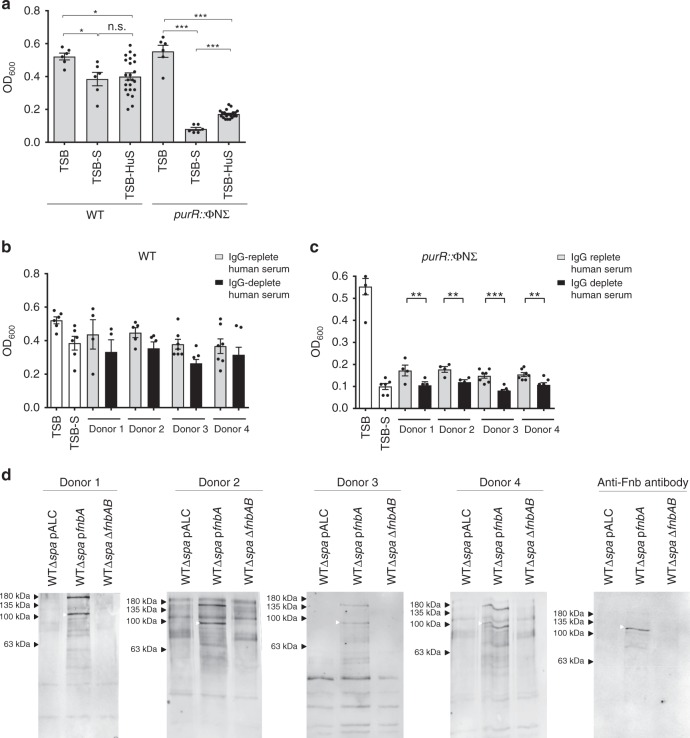
Anti-staphylococcal antibodies ameliorate *purR* hyper-clumping. **a** WT or the *purR*::ΦNΣ mutant were grown in TSB, TSB-S or TSB with 10% v/v fresh human serum (TSB-HuS) for 3 h and relative clumping ability was measured using OD_600_ as described above. Data shown are mean ± SEM of four independent experiments. *p-value < 0.05, **p-value < 0.01 and ***p-value < 0.001, based on a one-way ANOVA with a Bonferroni post-test. WT (**b**) or the *purR*::ΦNΣ mutant (**c**) were grown in TSB (white bars), TSB-S (white bars), TSB-HuS (grey bars) or TSB with IgG-depleted human serum (HuS) (black bars) for 3 h and relative clumping ability measured as above. Data shown are mean ± SEM of four experiments, with four donors. **p-value < 0.01, ***p-value < 0.001, based on a one-way ANOVA with a Bonferroni post-test. In **d**, whole cell lysates of WT, WT p*fnbA* or WTΔ*fnbAB* were used for western blots, with human serum (from donors in panels **b** and **c**) or a rabbit anti-Fnb serum (far right blot) used as a source of primary antibody. Source data are provided as a Source Data file

To date, despite the hypervirulence phenotype of *purR* mutation described above, no associations of *purR* mutations with human infection have been noted. A search of publicly available whole genome sequences identified a non-synonymous change or changes in the *purR* gene in 331 of 8201 sequences (Supplementary Data 1). However, few details on the infection type or outcome were available and, at this point, no correlations could be drawn between the presence of a *purR* mutation and disease severity. To begin to explore this further in the laboratory setting, we tested whether human serum can support hyper-clumping of *purR*::ΦNΣ bacteria. We isolated fresh human serum from healthy volunteers and this serum was used to assay for clumping as described above. When the WT and *purR*::ΦNΣ mutant were grown in TSB with 10% v/v human serum (TSB-HuS), the *purR*::ΦNΣ clumped but clumping was less pronounced than that seen in horse serum (Fig. 5a). Since the clumping phenotype relies on FnbA and FnbB, and their interaction with Fn, we hypothesised that anti-FnBP (i.e. blocking) antibodies are present in human serum, since humans are exposed to *S. aureus* throughout their lifetime, and that these antibodies would interfere with clumping. To test this, we passed human serum through a protein A column to remove IgG. TSB containing 10% v/v IgG-depleted human serum showed increased levels of clumping for the *purR*::ΦNΣ mutant, when compared to TSB-HuS, but no significant difference was observed for the WT (Fig. 5b, c). Moreover, addition of the purified human IgG to the *purR*::ΦNΣ mutant growing in TSB-S resulted in significant reduction of the clumping phenotype (Supplementary Figure 6). This indicates that antibodies present in human serum can interfere with the *purR*-dependent clumping phenotype. Western blot analysis determined that human serum did indeed contain anti-FnbA/B antibodies (Fig. 5d); while no signal could be detected for the WT, likely due to low expression, which is in agreement with our luminescence findings (Fig. 3c), bands could be visualised if a strain carried an *fnbA* overexpression construct. These data indicate that humans carry anti-FnbA/B antibodies that, while not necessarily protective against *S. aureus* infection, may confer protection against the hyper-virulent *purR* phenotype.

### Anti-FnBP antibodies protect against *purR* hypervirulence

**Fig. 6:**
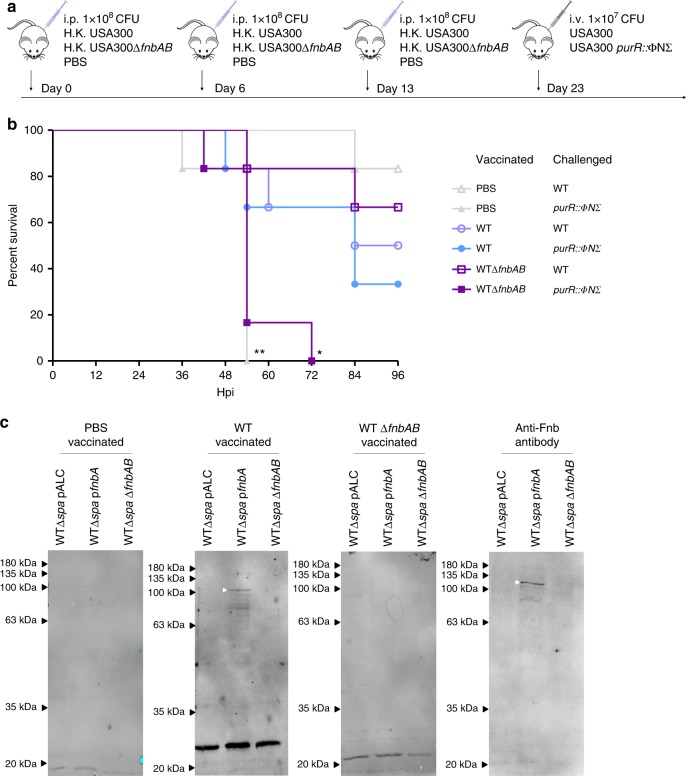
Vaccination with *S. aureus* expressing FnbAB is protective against a challenge with a *purR* mutant. **a** Vaccination scheme, with six animals per group. **b** Survival of animals challenged with 1 × 10^7^ CFU of WT or *purR*::ΦNΣ *S. aureus* following vaccination, as outlined in **a**. *p-value < 0.05, **p-value < 0.01, based on a Mantel-Cox test, as compared to WT vaccinated, *purR*::ΦNΣ challenged animals. **c** Whole cell lysate of WT, WT p*fnbA* or WTΔ*fnbAB* were used for a western blot, with serum from vaccinated animals or a rabbit anti-Fnb serum (far right) used as a primary antibody

Given our data indicated that anti-FnbA/B antibodies present in human serum can impair *purR* mutant clumping, we hypothesised that mice with antibodies recognizing the FnBPs would be protected from hypervirulence associated with *purR*::ΦNΣ infection. To test this, we vaccinated groups of 12 mice intraperitoneally with either 1 × 10^8^ heat-killed (HK) USA300, 1 × 10^8^ HK USA300Δ*fnbAB*, or with PBS on day 0, 6 and 13 (Fig. 6a). On day 23, animals in each group were challenged with either live WT USA300 or USA300 *purR*::ΦNΣ bacteria. In groups vaccinated with WT USA300, significantly more animals survived challenge with the *purR*::ΦNΣ strain, when compared to those vaccinated with USA300Δ*fnbAB* or the vehicle control (Fig. 6b). Serum from vaccinated animals demonstrated that mice receiving HK WT USA300 raised antibodies towards *S. aureus* antigens, including FnbA/B, while those challenged with HK USA300Δ*fnbAB* likewise raised antibodies to many antigens, but not to FnbA/B proteins (Fig. 6c), indicating the protective response is indeed due to anti-FnbA/B antibodies.

## Discussion

*Staphylococcus aureus* is a prolific pathogen of humans and animals and, with the emergence of multi-drug resistant strains, efforts to uncover novel drug targets and deliverables have intensified. The discovery and characterisation of novel drug targets often necessitates genetic manipulation of the pathogen under investigation, yet during commonly employed mutagenesis procedures off-target mutations can be induced that profoundly influence the pathogenic potential of this bacterium. Here, we found that exposure of *S. aureus* to stress such as elevated temperatures (i.e. 42 °C during mutagenesis) or an active host immune response can induce loss of function mutations in the *purR* gene. PurR is a predicted transcriptional regulator of the purine biosynthetic operon as in other bacteria^28,29^, and expression of the *pur* operon is elevated in *S. aureus purR* mutants. The observation that exposure to stress induces *purR* mutations could suggest that increased purine biosynthesis is of benefit to *S. aureus* under these conditions. In *E. coli* exposure to elevated pH (i.e. pH ≥ 9.0) is also reported to induce *purR* mutations consistent with the notion that de-repression of the *pur* operon may promote bacterial survival during exposure to certain stresses^30^. Remarkably, inactivation of *purR* in *S. aureus* causes robust bacterial clumping in the presence of the extracellular matrix protein fibronectin, and causes hyper-virulence in a murine model of systemic infection. Interest in purine metabolism in *S. aureus* has emerged because disruption of purine biosynthetic pathways can yield growth defects and diminish virulence^31–33^, however, the contribution of the PurR regulator to the physiology of *S. aureus* has not, to our knowledge, been explored. Consistent with reports of reduced *S. aureus* fitness when purine biosynthesis is disrupted, we find that provision of PurR *in trans* reverses the enhanced virulence of *purR*::ΦNΣ *S. aureus* USA300 and attenuates infection (see Fig. 5a). Presumably, overexpression of PurR represses the *purEKCSQLFMNHD* biosynthetic operon, thereby mimicking the effects of a *pur* gene deletion, which renders *S. aureus* less able to grow in human blood and during infection^34–36^.

How purine metabolism impacts *S. aureus* is unclear, but one possibility is that it involves the bacterial stringent response, which could conceivably enhance resistance to stress. In *S. aureus* the stringent response can be induced upon amino acid starvation and exposure to the antibiotic mupirocin^37^, and to the cell wall-targeting antibiotic vancomycin^38^. Interestingly, exposure to mupirocin and activation of the stringent response has been shown to repress expression of multiple Pur proteins such as PurE, PurQ, and PurK and have a more global impact on gene expression^39^. Contrary to these findings, our RNAseq data revealed that *purR*::ΦNΣ *S. aureus* demonstrated upregulation of *pur* genes, but no global changes in gene expression, arguing that deletion of the *purR* gene in *S. aureus* is unlikely to evoke the stringent response.

The hypervirulent phenotype of *purR*::ΦNΣ *S. aureus* is dependent on expression of fibronectin binding proteins A and/or B (FnbA/B) and deletion of the tandemly-duplicated *fnbAB* genes ablates lethality of the *purR*::ΦNΣ mutant. FnbA/B are known to bind the extracellular matrix proteins fibronectin and fibrinogen, and contribute to the adhesion of wild-type *S. aureus* to host tissues, to mediate cellular invasion, and promote virulence during infection^40–42^. Our analysis of *fnbA* and *fnbB* gene expression reveals that PurR plays a heretofore undescribed role in repressing FnbA/B expression. Our data support the notion that PurR negatively regulates FnBP expression in early log-phase cells and de-repression of FnBP transcription in *purR* deficient bacteria occurs only transiently, indicating additional regulatory factors also coordinate FnBP expression. Indeed, previous work characterizing FnbA expression has demonstrated that the global regulators of staphylococcal virulence Agr and Sar coordinate FnbA expression^43,44^. Nevertheless, aberrant regulation of FnbA/B expression in *S. aureus purR* mutants plays a vital role in the hyper-agglutination and the hypervirulent phenotypes described herein. Furthermore, the importance of carefully regulated FnBP expression during *S. aureus* infection is also evidenced by experiments where constitutive expression of plasmid encoded *fnbA* causes lethal *S. aureus* infection irrespective of *purR* (Fig. 4f). At present it is unclear what mechanism within the host causes mortality. However, due to the fact that FnBP proteins promote bacterial clumping and/or interaction with host platelets^45,46^, it is tempting to speculate that thrombus formation may be a contributing factor. Our data also reveal that FnbA/B contribute significantly to colonization of kidney and liver tissue by *purR*::ΦNΣ *S. aureus* during systemic infection. This contrasts wild-type USA300 where FnbA/B expression seems to play a less important role, as deletion of the *fnbA/B* genes alone did not significantly affect colonization of the murine heart, kidney or liver (see Fig. 4g). Presumably this can be attributed, in part, to the multitude of virulence factors that *S. aureus* can express and their potential redundancy. The relative differences in the contribution of FnBP proteins to the pathogenesis of wild-type *S. aureus* USA300 and in the *purR*::ΦNΣ strain is also evident from our vaccination study, where immunization of mice with FnbA/B-expressing *S. aureus* conferred protection against *purR*::ΦNΣ lethality but was largely without effect on wild-type USA300 infection.

The differences observed in *purR*::ΦNΣ bacterial clumping in human versus horse serum prompted our consideration for the existence of anti-staphylococcal antibodies directed to the FnBPs of *S. aureus*, as has previously been reported^47,48^. Human sera do contain naturally occurring anti-FnbA/B antibodies and the presence of these antibodies correlates with reduced *purR* bacterial clumping in human serum (see Fig. 5). Importantly, these data suggest that some of these naturally occurring antibodies neutralize FnBP-Fn interactions, and the presence of these immunoglobulins may confer protection against the potentially severe consequences of infection with *S. aureus purR* variants that may arise in humans. Several attempts have been made to create an anti-*S. aureus* vaccine however, to date, no vaccine has successfully protected against human infection^49–52^. Despite this, it is conceivable that immunization with certain *S. aureus* antigens may offer protection against aspects of staphylococcal pathogenesis (e.g. FnbA/B-dependent events) without altogether preventing infection, a notion that is supported by our data. Nevertheless, the pursuit of a vaccine that will protect against *S. aureus* infection over and above naturally occurring humoral immunity represents an important aim that will likely require a better understanding of human infection by *S. aureus*.

In summary, we have discovered that mutations in the *S. aureus purR* gene, arising from exposure of *S. aureus* to routine genetic manipulation or to the murine infection environment, can profoundly impact the pathogenesis of this bacterium. Hypervirulence associated with strains carrying *purR* mutations would undoubtedly offset the potentially desired attenuation phenotype associated with the mutation under study, severely confounding the interpretation of results of *S. aureus* virulence studies. As such, we urge *S. aureus* investigators to judiciously characterize mutant strains for off-target mutations affecting toxin production (e.g. *sae*, as previously reported) and *purR*, the latter easily tested by growing strains in media containing horse serum. The use of next generation sequencing makes identifying these and other off-target mutations straight forward. Our data show that the mechanism of *purR* mutant hypervirulence occurs via increased expression of fibronectin binding proteins at low cell densities. The detailed in vivo events occurring during infection of mice with *S. aureus purR* mutants are currently under study in our laboratory.

## Methods

### Bacterial growth conditions

Bacterial strains and plasmids used in this study are listed in Table 1 and primers are listed in Table 2. *E. coli* was grown in Luria-Bertani (LB) broth and *S. aureus* was grown in tryptic soy broth (TSB) at 37 °C, shaken at 200 rpm, unless otherwise stated. Where appropriate, media were supplemented with erythromycin (3 µg/mL), chloramphenicol (12 µg/mL), lincomycin (10 µg/mL), ampicillin (100 µg/mL) or tetracycline (3 µg/mL). Solid media were supplemented with 1.5% (w/v) Bacto agar.

**Table 1:** Bacterial strains used in this study

Strain	Description	Source
*S. aureus*		
USA300 LAC	CA-MRSA; cured of resistance plasmids	Lab stock
RN4220	r_K_^−^ m_K_^+^; capable of accepting foreign DNA	Lab stock
SH1000	WT *S. aureus* strain derived from 8325-4	Lab stock
SH1000 *purR*::ΦNΣ	Strain SH1000 containing a transposon insertion in the *purR* gene	This study
SH1000 *purR*::ΦNΣ + p*purR*	Strain SH1000 containing a transposon insertion in the *purR* gene and a plasmid carrying a full-length *purR* gene	This study
RN6390	WT *S. aureus* strain	Lab stock
RN6390 *purR*::ΦNΣ	Strain RN6390 containing a transposon insertion in the *purR* gene	This study
RN6390 *purR*::ΦNΣ + p*purR*	Strain RN6390 containing a transposon insertion in the *purR* gene and a plasmid carrying a full-length *purR* gene	This study
MN8	WT *S. aureus* strain	Lab stock
MN8 *purR*::ΦNΣ	Strain MN8 containing a transposon insertion in the *purR* gene	This study
MN8 *purR*::ΦNΣ + p*purR*	Strain MN8 containing a transposon insertion in the *purR* gene and a plasmid carrying a full-length *purR* gene	This study
Newman	WT *S. aureus* strain	Lab stock
Newman *purR*::ΦNΣ	Strain Newman containing a transposon insertion in the *purR* gene	This study
Newman *purR*::ΦNΣ + p*purR*	Strain Newman containing a transposon insertion in the *purR* gene and a plasmid carrying a full-length *purR* gene	This study
USA300 *purR*::ΦNΣ	Strain USA300 LAC containing a transposon insertion in the *purR* gene	This study
USA300 *purR*::ΦNΣ + p*purR*	Strain USA300 LAC containing a transposon insertion in the *purR* gene and a plasmid carrying a full-length *purR* gene	This study
USA300 Δ*fnbAB*	Strain USA300 LAC with a complete deletion of the *fnbA* and *fnbB* genes	This study
USA300 *purR*::ΦNΣ Δ*fnbAB*	Strain USA300 LAC with a complete deletion of the *fnbA* and *fnbB* genes and a transposon insertion in the *purR* gene	This study
USA300 Δ*spa*Δ*sbi*	Strain USA300 with a complete deletion in the *spa* and *sbi* genes	This study
USA300 Δ*spa*Δ*sbi* Δ*fnbAB*	Strain USA300 LAC with a complete deletion of the *fnbA* and *fnbB* genes and the *spa* and *sbi* genes	This study
USA300 Δ*spa*Δ*sbi purR*::ΦNΣ	Strain USA300 with a complete deletion in the *spa* and *sbi* genes and a transposon insertion in the *purR* gene	This study
USA300 Δ*spa*Δ*sbi* Δ*fnbAB purR*::ΦNΣ	Strain USA300 LAC with a complete deletion of the *fnbA* and *fnbB* genes and the *spa* and *sbi* genes and a transposon insertion in the *purR* gene	This study
USA300 *purR*^R96A^	Strain USA300 LAC with a R96A SNP in the *purR* gene	This study
USA300 *purR*^Q52P^	Strain USA300 LAC with a Q52P SNP in the *purR* gene	This study
USA300 *purR*^Q240Stop^	Strain USA300 LAC with a Q240Stop SNP in the *purR* gene	This study
USA300 *purR*^S172Stop^	Strain USA300 LAC with a S172Stop SNP in the *purR* gene	This study
USA300 *purR*^V156E^	Strain USA300 LAC with a V156E SNP in the *purR* gene	This study
USA300 *purR*^V229frameshift^	Strain USA300 LAC with a V229 frameshift SNP in the *purR* gene	This study
*E. coli*		
DH5α	FΦ80*lacZ*ΔM15Δ(*lacZYAargF)*u169 *recA*1 *endA* 1 *hsd*R17 (rK,mK^+^) *phoA sup*E44λ*thi*1 *gyrA*96 *relA* 1	Promega

**Table 2:** Primers used in this study

Primer name	Primer sequence
PurR F	TTTGGTACCATATCTTGAAAAGTGGTGCAGATGG
PurR R	TTTGAGCTCCCTGCTTCTTCCAAAACAACCTTTA
pALC MCS F	ATACCGCACAGATGCGTAAGG
pALC MCS R	CGATGACTTAGTAAAGCACATCTAA
FnbAB Up F	GGGGACAAGTTTGTACAAAAAAGCAGGCTCACAGATACTTCCAAGATTCTCAAACC
FnbAB Up R	GGACCTCCGCGGCAGTGGAACAAGGTAAAGTAGTAACAC
FnbABDown F	GGACCTCCGCGGGTATTCAAGTCATCAGAAACCCTTGTC
FnbABDown R	GGGGACCACTTTGTACAAGAAAGCTGGGTCAGGGCCTATATTTAACAAAGTTGCAC
pGYluxFnbA F	GCGCCCGGGGCAATATATTGCCTTGAAACACG
pGYluxFnbA R	GCGGTCGACTATAATATCTCCCTTTAAATGC
pGYluxFnbB F	GCGCCCGGGGTGTTTTCTGATTGCTTCATTGC
pGYluxFnbB R	GCGGTCGACTATAATATTCTCCCTTAAATGC
PurE F	GGGCAGTTCTTCCGATTGGA
PurE R	CTGTTCGCCCTTGACTGCTA
FnbA F	TTTGGATCCTGTGCGTATTGTACAGGCGA
FnbA R	TTTGAGCTCAGCCGTATTTCAAGCCGACA
qPCR PurE F	CTTCTGAAGCGAGAGAAAGAGGTATAA
qPCR PurE R	CAATAACTGGTAGCGTCGTTAATGATG
qPCR FnbA F	CGGCATTAGAAAACATAAATTGGG
qPCR FnbA R	GTTTTATTATCAGTAGCTGAATTCCC
qPCR FnbB F	GAAAACACAAATTGGGAGCG
qPCR FnbB R	TGTTTCGCTTGCTTTACTTTC

### PCR and construct generation

*S. aureus* strain USA300 LAC, cured of the 27-kb plasmid that confers antibiotic resistance, was used as the WT strain for mutant generation, unless otherwise stated. For mobilizing transposon insertion mutations into various genetic backgrounds, phage transduction was performed according to standard techniques. Phage lysate was prepared from the donor strain using phage 80α, recipient strains were infected and transductants selected using appropriate antibiotics. Insertions were confirmed by PCR. Markerless deletions were constructed using the pKOR1 system, as previously described^53^. Briefly, upstream and downstream regions flanking the FnbAB genes were amplified with primers FnbAB Up F and Up R, and FnbAB Down F and Down R, respectively, using Phusion DNA polymerase and recombined into pKOR1. The resulting vector was passaged through RN4220 and subsequently introduced into strains of interest by electroporation. Genomic deletions were confirmed by PCR with primers hybridizing outside of the cloned area of interest. The *purE* promoter-glucuronidase fusion reporter was synthesised by Integrated DNA Technologies (IDT, Canada), and ligated into pLL29. pLL29 was transformed into RN4220 containing a plasmid encoding an integrase and later transduced into USA300 and derivatives^54^. For complementation with WT *purR* or *fnbA*, the full-length genes were amplified using primers PurR F and PurR R and FnbA F and FnbA R, respectively, ligated into pALC2073 and recombinant plasmids transformed into *E. coli*. Plasmids were then passaged through RN4220, prior to transformation into the strain of interest. For insertion of *fnb* promoters into pGYlux, sequences were amplified from the USA300 genome with primer pairs pGYluxFnbA F and pGYluxFnbA R (for pGY:*fnbA)* and pGYluxFnbB F and pGYluxFnbB R (for pGY::*fnbB)*, respectively. Constructs were passaged through RN4220, prior to transformation into the strain of interest.

### Clumping assays

For measurement of clumping in serum (horse or human), overnight cultures in TSB were diluted to OD_600_ 0.03 in 2 mL TSB or TSB with 10% (v/v) serum in a 13 mL tube and grown at 37 °C, with shaking at 200 rpm for 3.5 h. Cultures were allowed to sit without shaking for 5 min and the OD_600_ of the middle of the culture was determined. The same cultures were imaged live on a brightfield Leica microscope at ×40 magnification.

### Fibronectin removal

To remove fibronectin from horse serum, sterile, heat-inactivated horse serum was passaged over a column of gelatin sepharose (GE healthcare) (column bed volume of 5.5 mL) at ~1 mL/min and the flow through collected. The column was washed with ~20 mL of phosphate-buffered saline (PBS) and bound fibronectin was eluted with PBS + 4 M urea. The column was re-generated as per manufacturer’s instructions and the run-through from the first purification passaged again. A total of three passages over the column were performed and the fibronectin-free serum was sterilized by passage through a 0.22 µm filter. The different run troughs were used at 10% (v/v) in standard clumping assays, as described above.

### Electron microscopy

*S. aureus* strains were grown in TSB or TSB with 10% horse serum for 3.5 h, as previously described for clumping assays. The bacteria were then fixed overnight with a modified Karnovsky’s fixative (2.5% glutaraldehyde (v/v) + 2% paraformaldehyde (v/v) in 0.1 M cacodylate buffer, pH 7.2). The fixed bacteria were embedded in a 1% (w/v) agarose suspension and post-fixed with 1% (w/v) osmium tetroxide for 2 h, followed by a 2-h en bloc 0.5% (w/v) uranyl acetate strain. Samples were then progressively dehydrated with 15-min treatments of increasingly concentrated ethanol solutions (50, 70, 90, 95, 100% (v/v)). After dehydration, the samples were embedded in Epon-Araldite and ultrathin sections (70 nm) were cut and placed on nickel grids using an Ultracut microtome. The cut samples were surface stained for 15 min with 0.5% (w/v) uranyl acetate and viewed with a Phillips 420 transmission electron microscope equipped with a Hamamatsu Orca 2 MPx HRL camera.

### Preparation of proteins

For examination of secreted protein profiles, strains were grown to the desired OD_600_ in TSB and normalised to OD_600_ of 6.0, pelleted by centrifugation, and supernatant mixed with 100% ethanol at a 1:3 ratio. Samples were incubated at −20 °C for 4–8 h and proteins pelleted at 5000 × *g* for 30 min at 4 °C. Pellets were re-suspended in 1:20th of the original cultured volume in PBS and stored at −20 °C. For whole cell lysate preparation, cells grown to the desired density were pelleted, washed once with PBS, re-suspended in 1:20th of the original volume in PBS with 400 µg lysostaphin and incubated at 37 °C for 1 h. Samples were passaged twice through a Cell-Disruptor (Constant Systems Ltd.) at 34,000 p.s.i., pelleted at 5000 × *g* for 10 min and supernatant harvested. For mass spectrometry analysis of bacterial clumps, the *purR::ΦΝΣ* mutant strain was grown in TSB with 10% (v/v) for 3 h at 37 °C, and clumps allowed to settle at the bottom of the tube. Clumps were washed three times with PBS, dissolved in 1% SDS at 55 °C for 1 h and run on a 7% SDS polyacrylamide gel. Bands of interest were picked for LC-MS-MS analysis.

### Western blots

Strains used for Western blot analysis were the same as described above, with the additional deletion of protein A (*spa)* and *sbi*, to eliminate non-specific IgG interactions. Whole cell lysate was prepared as described above, mixed with 1× Laemmli buffer (60 mM Tris-HCl, pH 6.8, 2% (w/v) SDS, 10% (v/v) glycerol, 5% (v/v) β-mercaptoethanol, 0.01% (w/v) bromphenol blue), boiled for 10 min and separated on a 10% polyacrylamide gel. Following electrophoresis, proteins were transferred to a nitrocellulose membrane following standard protocols. Human, mouse or horse sera (1:500 dilution) or rabbit anti-FnbA antiserum^55^ (1:500 dilution) were used as a primary antibody, and secondary antibody (conjugated to IRDye 800; Li-Cor Biosciences, Lincoln, NE) was used at a 1:20,000 dilution. Membranes were scanned on a Li-Cor Odyssey Infrared Imager (Li-Cor Biosciences) and visualized using Odyssey Version 3.0 software.

### Biofilm assay

Biofilm assays were performed as described previously^56^. Briefly, 200 µL of TSB supplemented with 0.4% w/v glucose was inoculated with a 1:100 dilution of an overnight culture. After static incubation at 37 °C for 16–22 h, cells were washed three times with PBS and fixed by drying at 42 °C. Crystal violet (0.4% (w/v)) was used to stain cells for 15 min, before being dissolved in glacial acetic acid (10% (v/v)) and level of adhesion quantified by absorbance at 595 nm. Absorbance was normalized to the WT strain, which was set to 1.

### Luciferase-based measurements of *fnb* promoter activity

WT or *purR*::ΦNΣ strains carrying pGYlux constructs with the promoter of *fnbA* (pGY::*fnbA*) or *fnbB* (pGY::*fnbB*) were used. For luciferase measurements, overnight cultures grown in TSB with chloramphenicol were diluted to OD_600_ of 0.01 in TSB with chloramphenicol and 200 µl added to a white optical 96-well plate (Thermo Fisher). Growth and luminescence were measured in a BioTek Synergy H4 plate reader at 37 °C with shaking. Data for both absorbance and luminescence was normalised to blank measurements for each time point.

### RNA extraction and RNAseq

*S. aureus* strains were grown overnight, subcultured to an OD_600_ equivalent of 0.01 in TSB and grown to the desired growth phase. Cells equating to an OD_600_ of 3.0 were harvested for each culture, and RNA extraction was performed by E.Z.N.A® total RNA kit (Bio-Rad) according to the manufacturer’s instructions with the addition of 0.25 μg/mL lysostaphin to the lysis solution. RNA purity was determined by visualisation on an agarose gel, and RNA concentration was determined by NanoDrop® ND-1000 UV-Vis spectrophotometer. cDNA preparation was performed using 500 ng of total cellular RNA reverse-transcribed using Superscript^TM^ II reverse transcriptase (Invitrogen) according to the manufacturer’s instructions. For each qPCR, 1 μg of cDNA was amplified in a Rotor-Gene 6000 (Corbett Life Science) using the iScript One-Step RT-PCR kit with SYBR Green (Bio-Rad). Gene expression for each sample was quantified in relation to *rpoB* expression. A standard curve was generated for each gene examined. For RNAseq, RNA was extracted as above, a library was constructed using an Illumina Script Seq RNA sequencing kit and sequenced on an Illumina MiSeq.

### Genome sequence analysis

The nucleotide sequence of the *purR* gene was downloaded from 8207 *S. aureus* available genome sequences or assemblies from the NCBI database (1st Dec 2017). Sequences were translated and amino acids aligned to the USA300 LAC *purR* sequence using MEGA 7. For strains with sequence changes as compared to USA300 FPR3757, the information available was compiled into Supplementary Data 1.

### Ethics statements

Human blood was obtained from healthy adult volunteers, with written permission and in compliance with protocol 109059 approved by the Office of Research Ethics at the University of Western Ontario. All animal experiments were performed in compliance with guidelines set out by the Canadian Council on Animal Care. All animal protocols (protocol 2017-028) were reviewed and approved by the University of Western Ontario Animal Use Subcommittee, a subcommittee of the University Council on Animal Care.

### Human serum antibody removal

Blood was allowed to clot for 30 min at RT, centrifuged at 400 × *g* for 10 min (no brake) and serum harvested. Serum was filtered through a 0.22 µm filter and heat-inactivated for 1 h at 56 °C. For removal of antibody, 4 mL serum was loaded on a HiTrap protein A column (GE healthcare) at 1 mL/min, followed by a 15 min wash (20 mM sodium phosphate, pH 7) at 1 mL/min. Antibody was eluted (0.1 M sodium citrate, pH 4) in three fractions (1.5 mL/each) at 1 mL/min. Serum used for clumping assays was passaged through the column twice. Eluted IgG was filtered through a 0.22 µm filter, concentrated with an Amicon ultra-50 centrifugal filter and added to clumping assays containing 10% (v/v) horse serum.

### Mouse infections

Six- to eight-week-old female BALB/c mice (Charles River laboratories) were injected via tail vein with 100 µL of bacterial culture, containing 1 × 10^6^–1 × 10^7^ CFU of bacteria, as described in the text. To prepare the bacteria, strains were grown to OD_600_ 2–2.5 in TSB, washed twice with PBS and re-suspended to the desired OD_600_ in PBS. Infections were allowed to proceed for up to 96 h before animals were euthanized, or when they met guidelines for early euthanasia. Organs were harvested in PBS + 0.1% Triton X-100 (Sigma), homogenised in a Bullet Blender Storm (Next Advance, Troy, NY), using two runs of 5 min at setting 10, and metal beads. Dilutions of organ homogenates were plated on TSA for CFU enumeration. For vaccination studies, bacteria were grown to OD_600_ of ~0.6, bacteria washed as above, heat killed at 85 °C for 15 min and 100 µL, equivalent to ~1 × 10^8^ CFU, were injected intraperitoneally (IP). For challenge post vaccination, infections were as outlined above.

### Statistical analysis

Statistical analyses were performed with GraphPad Prism software v5.0 or v7.0.

### Reporting Summary

Further information on experimental design is available in the Nature Research Reporting Summary linked to this Article.

## Supplementary Information

Supplementary Information

Description of Additional Supplementary Files

Supplementary Data 1

Reporting Summary

Source Data

## Data Availability

A Reporting Summary for this Article is available as a Supplementary Information file. The source data underlying Figs. 1b, 2b-d, 3c-g, 4c-d, 4g, 5a-c, Supplementary Figures 1 b-d, 2 a-d, 4d and 5 are provided as a Source Data file. Whole genome sequence data have been deposited in the NCBI genbank nucleotide database under accession number PRJNA513342. RNAseq data has been deposited in the NCBI GEO database under accession number GSE124869. Other relevant data are available from the corresponding author upon request.
