# Supplementary material for: Stress-induced inactivation of the Staphylococcus aureus purine biosynthesis repressor leads to hypervirulence
Source: Nat Commun. 2019 Feb 15;10:775. doi: 10.1038/s41467-019-08724-x (PMC6377658; doi:10.1038/s41467-019-08724-x)
Supplement: Supplementary file 1 — Supplementary Information [file 41467_2019_8724_MOESM1_ESM.pdf]

Stress-induced inactivation of the *Staphylococcus aureus* purine biosynthesis repressor leads to hypervirulence

Goncheva *et al*

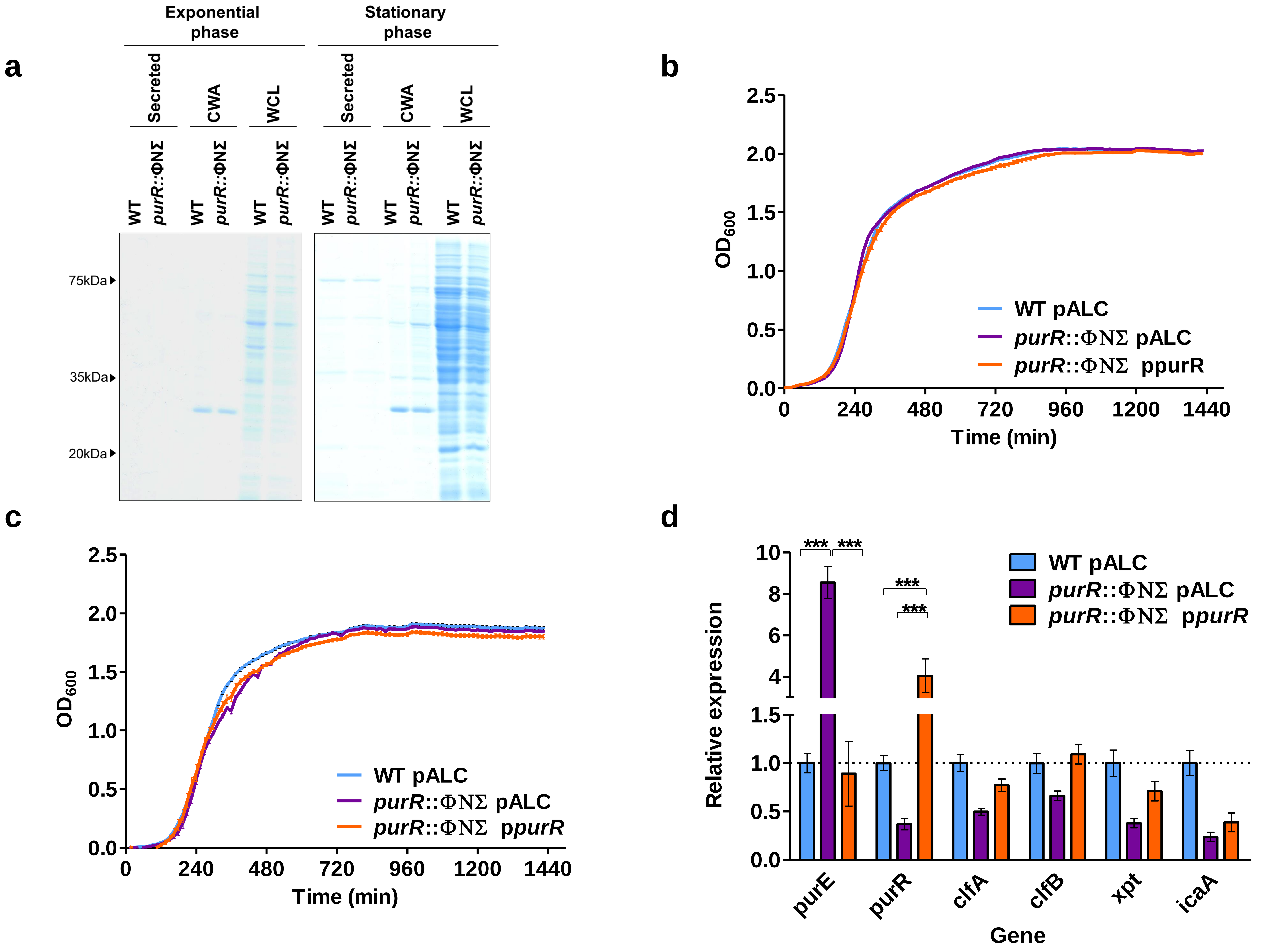

Supplementary Figure 1. Disruption of *purR* has minimal effect on the *S. aureus* proteome or growth. a, total protein of USA300 and USA300 *purR*::ΦNΣ grown to exponential (OD_{600} 0.6) or stationary phase (OD_{600} 6.0) and separated on a 12% SDS polyacrylamide gel. **b**, growth curves of USA300, USA300 *purR*::ΦNΣ or complemented *purR*::ΦNΣ mutant in TSB. **c**, growth curves of USA300, USA300 *purR*::ΦNΣ and complemented *purR*::ΦNΣ mutant in TSB-S. **d**, relative expression of a selection of genes following growth in TSB to OD_{600} of 1.0, measured by RT- PCR. All data were normalised to the levels of *rpoB* and the expression in the WT was set to 1.0. Data shown are mean ± SEM of 4 samples. *** indicates a p value < 0.001 based on a one-way ANOVA with a Bonferroni post-test. Source data are provided as a Source Data file.

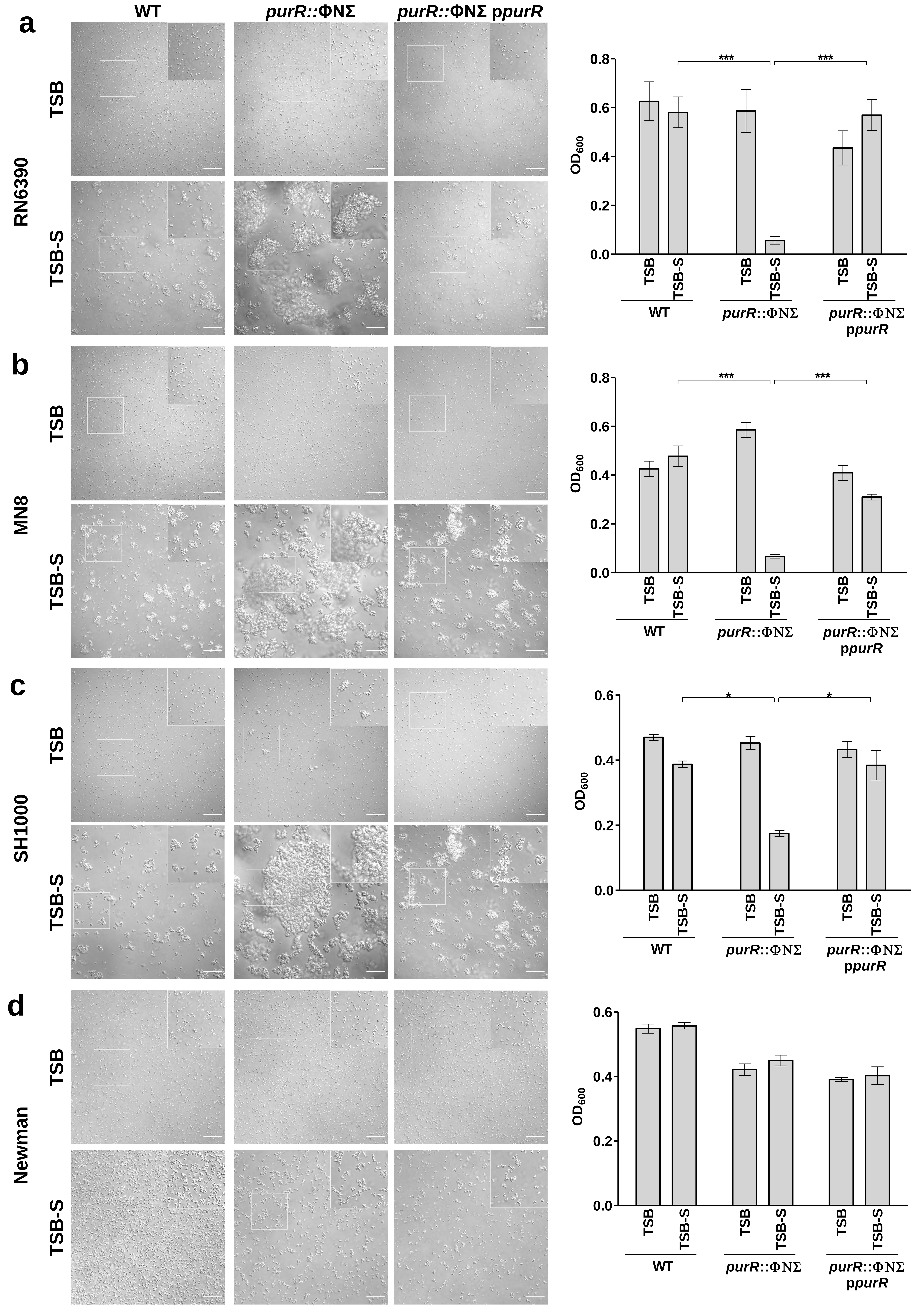

a
b
c
d
WT
purR::ΦΝΣ
purR::ΦΝΣ ppurR
RN6390
MN8
SH1000
Newman
TSB
TSB-S
OD600
0.0
0.2
0.4
0.6
0.8
***
*

Supplementary Figure 2. Disruption of *purR* results in a clumping phenotype in a variety of strains, but not in strain Newman. WT, *purR*::ΦΝΣ or *purR*::ΦΝΣ complemented constructs in strains RN6390 **(a),** MN8 **(b)**, SH1000 **(c)** or Newman **(d)** were grown in TSB or TSB-S for 3.5h. Cultures were imaged on a wide field microscope at 40x magnification (left panel) or absorbance measured (right panel). Bars equal 40µm. Data shown are mean ± SEM of 4 experiments. * indicates a p value < 0.05, *** indicates a p value < 0.001 based on a one-way ANOVA with a Bonferroni post-test. Source data are provided as a Source Data file.

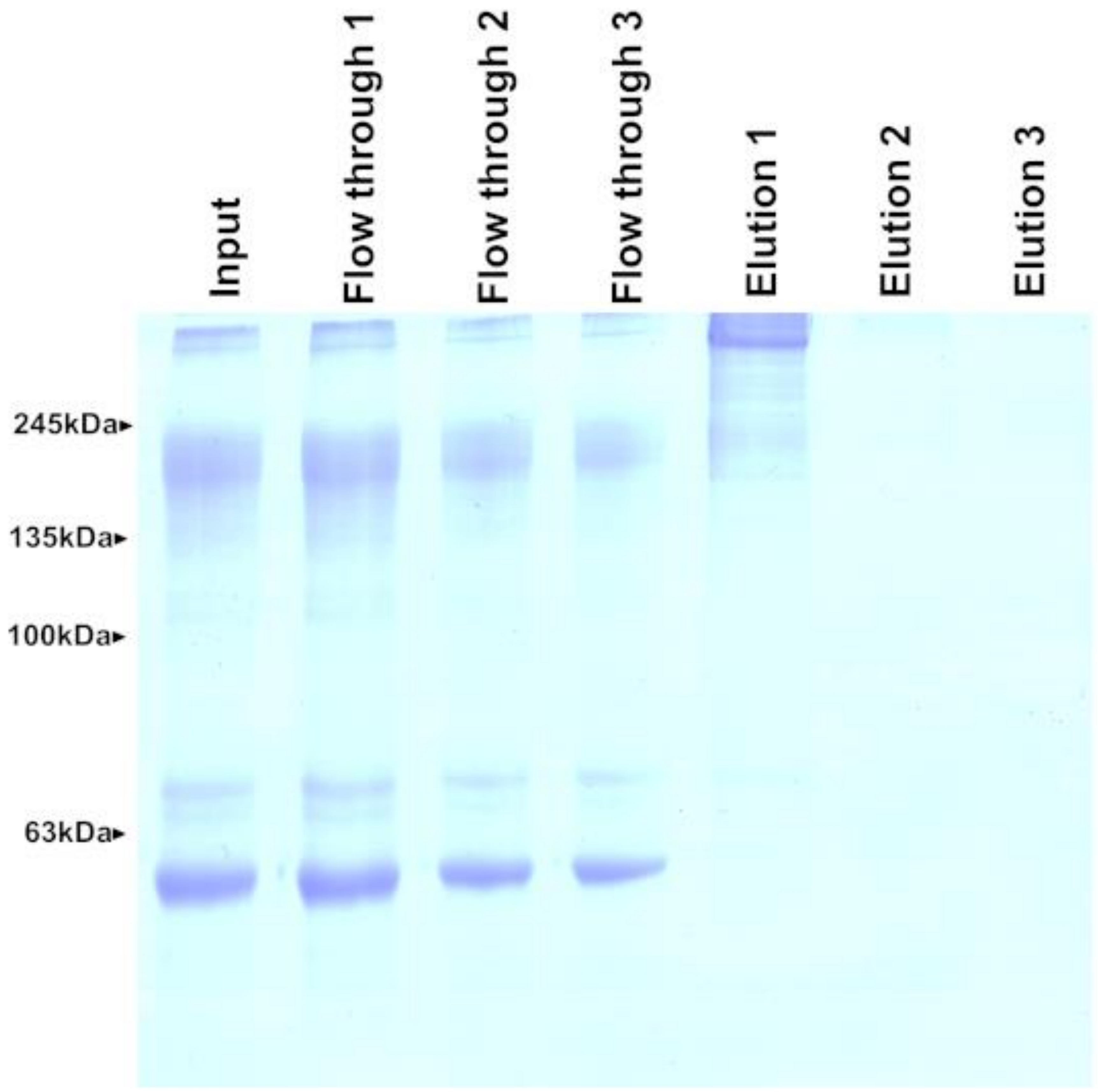

Supplementary Figure 3. Passage of horse serum over a gelatin column removes soluble fibronectin. Horse serum was passaged over a gelatin sepharose column 3 times. Column flow through and elutions were separated on a 7% SDS polyacrylamide gel.

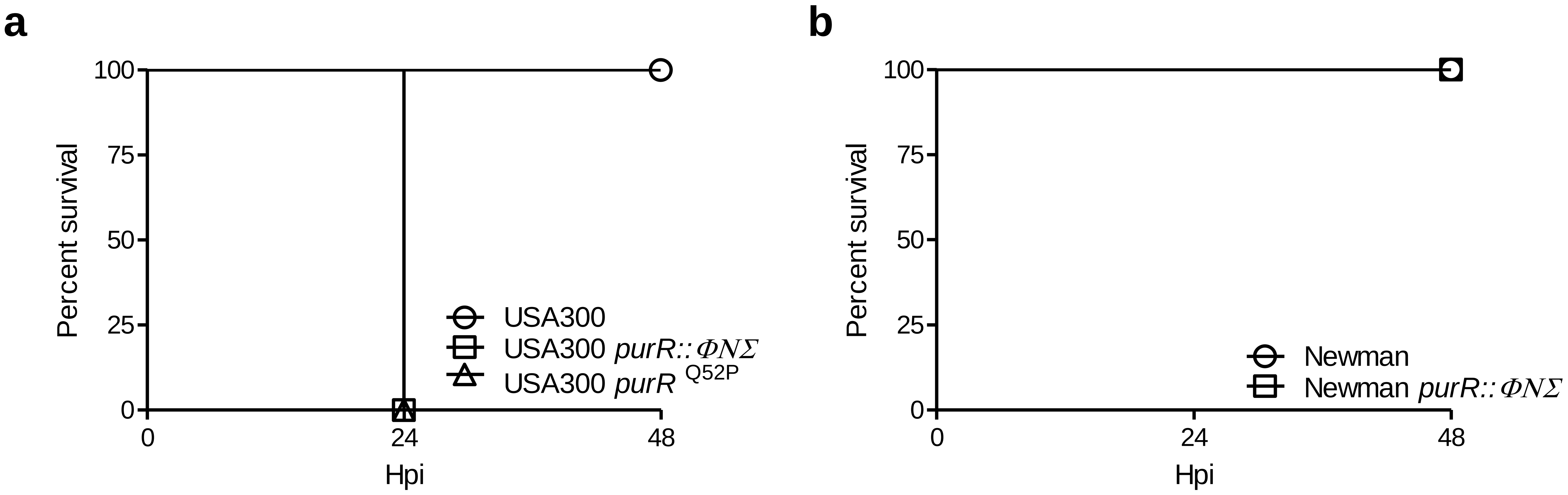

Supplementary Figure 4. A *S. aureus purR* SNP mutant is hypervirulent. a, animals were infected IV with $1x10^7$ CFU of USA300 WT, *purR*::ΦNΣ or *purR*^{Q52P} mutant and monitored over 48h. **b,** animals were infected IV with $1x10^7$ CFU of Newman WT or *purR*::ΦNΣ and monitored over 48h.

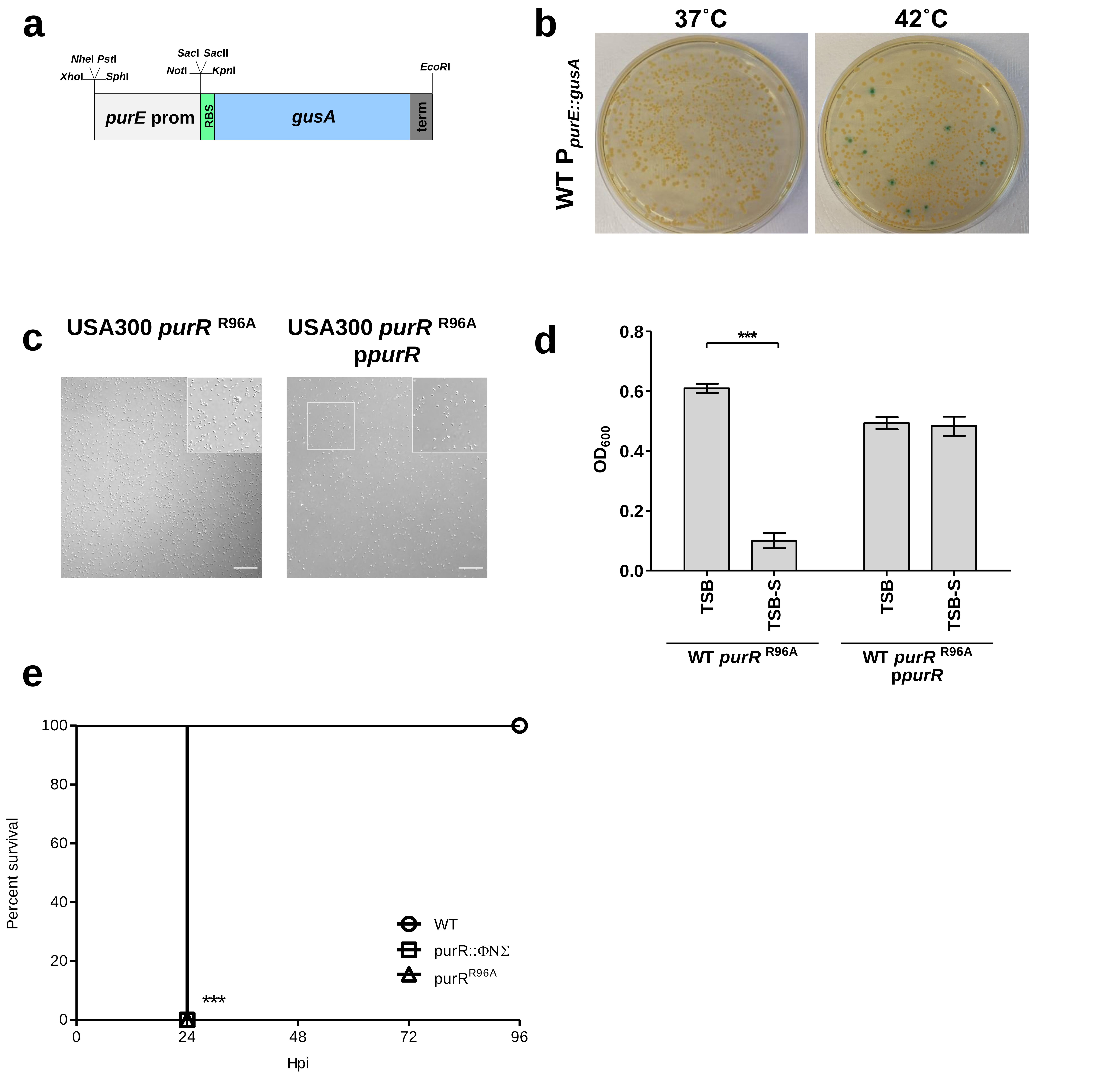

Supplementary Figure 5. Mutations in *purR* are selected for during growth at elevated temperatures and in vivo during infection of mice.a, schematic of the $P_{purE:gusA}$ construct that is integrated into the *S. aureus* genome. **b**, WT $P_{purE::gusA}$ after 5 passages at 37˚C (left) and 42˚C (right), grown on TSA with tetracycline and X-gluc. In **c**-**e**, characterization of a clone of *S. aureus* USA300 containing a *purR*^{R96A} SNP isolated from the kidney of a mouse infected for 4 days with WT USA300. Strains were grown in TSB or TSB-S for 3.5h, and cultures were imaged on a wide field microscope at 40x magnification **(c)** or relative clumping was measured using the OD_{600} assay described above **(d).** Data shown are mean ± SEM of 3 experiments. *** indicates a p value < 0.001 based on a one way ANOVA with a Bonferroni post test. In **(e),** animals were infected IV with $1x10^7$ CFU of WT, *purR*::ΦNΣ or *purR*^{R96A} mutant and monitored over 96h. *** indicates a p value < 0.001, based on a Mantel-Cox test. Source data are provided as a Source Data file.

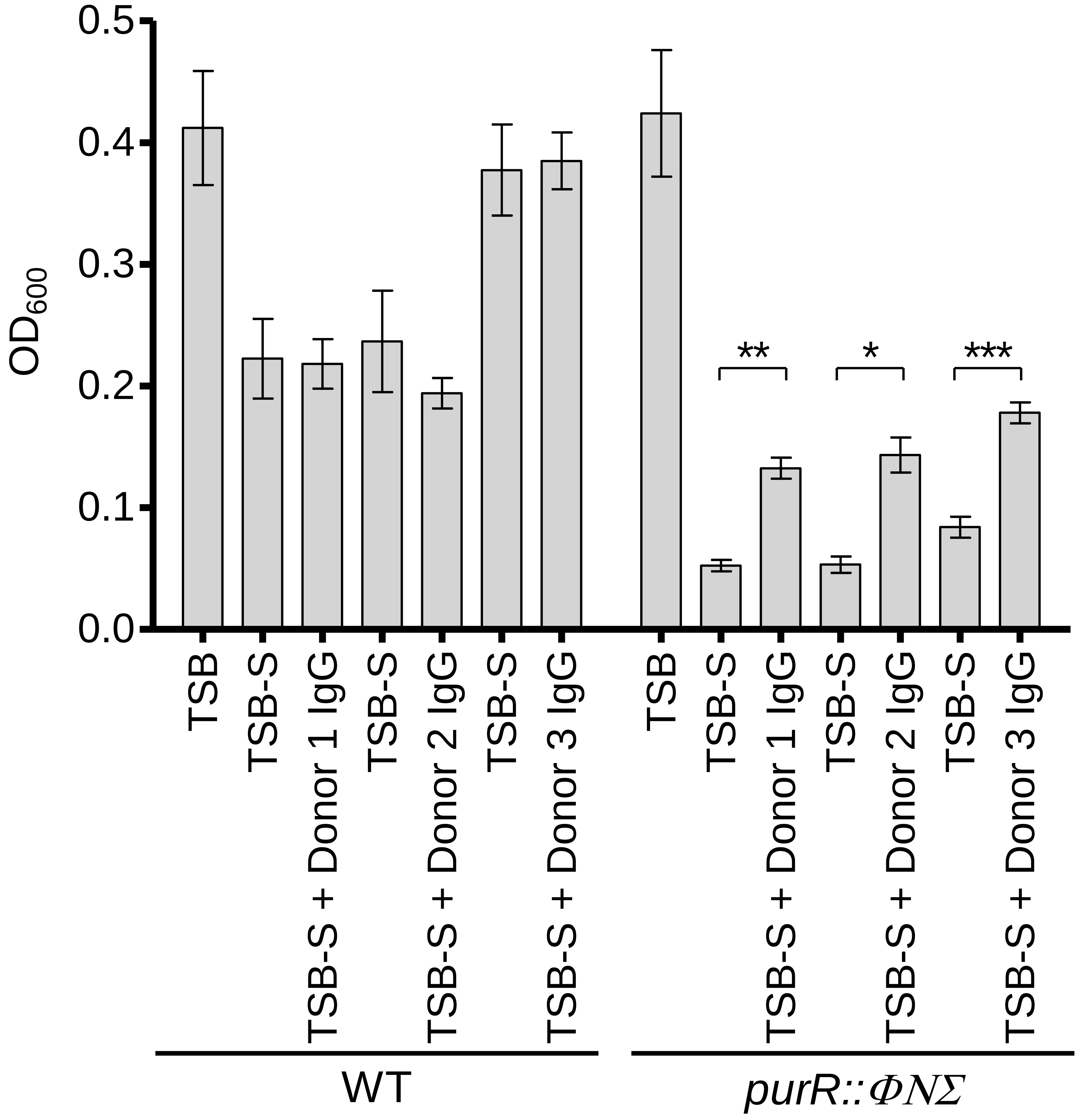

Supplementary Figure 6. Human IgG can alleviate *purR* dependent clumping in horse serum. Cultures of WT USA 300 or USA300 *purR*::ΦNΣ were grown in TSB, TSB-S or TSB-S with the addition of purified and concentrated human IgG (from serum IgG depletions shown in Figure 5). Cultures were allowed to grow for 3 h from a starting OD_{600} of 0.03 and the OD_{600} values of the center of liquid cultures after sitting for 5 min were determined. Data shown are mean ± SEM of 3-4 independent experiments with IgG from 3 different donors. * indicates a p value < 0.05, ** a p value < 0.01 and *** indicate a p value < 0.001, based on paired student t test. Source data are provided as a Source Data file.

Supplementary Table 1. Gene expression changes in a *purR*::ΦNΣ mutant, as measured by RNAseq.

Gene (Locus tag)	Log2 Fold change	P value
PurN (SAUSA300_0974)	4.2	0
PurH (SAUSA300_0975)	4.17	5.88E-13
PurQ (SAUSA300_0970)	4.13	0
PurC (SAUSA300_0968)	4.12	0
PurS (SAUSA300_0969)	4.04	0
PurM (SAUSA300_0973)	4.02	1.11E-16
PurL (SAUSA300_0971)	4	3.67E-12
PurD (SAUSA300_0976)	3.87	9.69E-14
PurK (SAUSA300_0967)	3.77	5.55E-16
PurF (SAUSA300_0972)	3.75	1.67E-15
PurE ((SAUSA300_0966)	3.64	2.55E-15
tRNA – Asn	2.63	0.08
tRNA – Ala	2.44	0.0003
tRNA – Ala	2.19	0.001
purB (SAUSA300_1889)	2.06	0.0001
purA (SAUSA300_0017)	1.85	2.37E-05
FnbA (SAUSA300_2441)	0.24	0.65
FnbB (SAUSA300_2440)	-1.81	0.001
clfA (SAUSA300_0772)	0.31	0.45
clfB (SAUSA300_2565)	-0.13	0.78
Xpt (SAUSA300_0386)	-0.69	0.4
icaA (SAUSA300_2600)	-4.71	0.0007

Supplementary Table 2. Quantification of lesion frequency 24 hpi. Mice were infected with 1x10^7 CFU of WT, *purR*::ΦNΣ or *purR*::ΦNΣ complemented strains for 24 h, organs were harvested, paraffin embedded, sectioned and stained with H&E and a Gram stain. Lesion numbers and severity were determined by a trained veterinary pathologist, blinded to experimental design. Severity scores were assigned as 0 = absent, 1 = mild, 2= moderate, 3 = severe. Data were collected from 2 animals per strain, with 2 sections per organ for each animal.

	Heart	Kidney	Liver	Spleen	Lung
	Frequency of lesions				
WT pALC	1	1	1	1	1
WT pALC	1	1	1	1	1
*purR::*ΦNΣ pALC	2	3	1	1	1
*purR::*ΦNΣ pALC	2	3	0	1	1
*purR::*ΦNΣ p*purR*	0	0	0	0	0
*purR::*ΦNΣ p*purR*	0	0	0	0	0
	Severity of lesions				
WT pALC	1	2	1	1	1
WT pALC	1	2	1	1	1
*purR::*ΦNΣ pALC	2.5	3	1	1	1
*purR::*ΦNΣ pALC	2.5	3	0	1	1
*purR::*ΦNΣ p*purR*	0	0	0	0	0
*purR::*ΦNΣ p*purR*	0	0	0	0	0
	Frequency x severity				
WT pALC	1	2	1	1	1
WT pALC	1	2	1	1	1
*purR::*ΦNΣ pALC	5	9	1	1	1
*purR::*ΦNΣ pALC	5	9	0	1	1
*purR::*ΦNΣ p*purR*	0	0	0	0	0
*purR::*ΦNΣ p*purR*	0	0	0	0	0
