# Supplementary material for: Stress-induced inactivation of the Staphylococcus aureus purine biosynthesis repressor leads to hypervirulence
Source: Nat Commun. 2019 Feb 15;10:775. doi: 10.1038/s41467-019-08724-x (PMC6377658; doi:10.1038/s41467-019-08724-x)
Supplement: Supplementary file 2 — Description of Additional Supplementary Files [file 41467_2019_8724_MOESM2_ESM.pdf]

Description of Additional Supplementary Files

File Name: Supplementary Data 1
Description: Table with the accession numbers, isolate type and the purR mutation type of S. aureus genomes that were found to have non-synonymous changes in purR.
