# Supplementary material for: Stress-induced inactivation of the Staphylococcus aureus purine biosynthesis repressor leads to hypervirulence
Source: Nat Commun. 2019 Feb 15;10:775. doi: 10.1038/s41467-019-08724-x (PMC6377658; doi:10.1038/s41467-019-08724-x)
Supplement: Supplementary file 4 — Reporting Summary [file 41467_2019_8724_MOESM4_ESM.pdf]

nature research

Corresponding author(s): D. E. Heinrichs

Last updated by author(s): Jan 9, 2019

Reporting Summary

Nature Research wishes to improve the reproducibility of the work that we publish. This form provides structure for consistency and transparency in reporting. For further information on Nature Research policies, see Authors & Referees and the Editorial Policy Checklist.

Statistics

For all statistical analyses, confirm that the following items are present in the figure legend, table legend, main text, or Methods section.

n/a	Confirmed	
☐	☒	The exact sample size (*n*) for each experimental group/condition, given as a discrete number and unit of measurement
☐	☒	A statement on whether measurements were taken from distinct samples or whether the same sample was measured repeatedly
☐	☒	The statistical test(s) used AND whether they are one- or two-sided<br>*Only common tests should be described solely by name; describe more complex techniques in the Methods section.*
☐	☒	A description of all covariates tested
☐	☒	A description of any assumptions or corrections, such as tests of normality and adjustment for multiple comparisons
☐	☒	A full description of the statistical parameters including central tendency (e.g. means) or other basic estimates (e.g. regression coefficient) AND variation (e.g. standard deviation) or associated estimates of uncertainty (e.g. confidence intervals)
☒	☐	For null hypothesis testing, the test statistic (e.g. *F*, *t*, *r*) with confidence intervals, effect sizes, degrees of freedom and *P* value noted<br>*Give P values as exact values whenever suitable.*
☒	☐	For Bayesian analysis, information on the choice of priors and Markov chain Monte Carlo settings
☒	☐	For hierarchical and complex designs, identification of the appropriate level for tests and full reporting of outcomes
☒	☐	Estimates of effect sizes (e.g. Cohen's *d*, Pearson's *r*), indicating how they were calculated

Our web collection on statistics for biologists contains articles on many of the points above.

Software and code

Policy information about availability of computer code

Data collection	No software used
Data analysis	GraphPad Prism version 5.0 or 7.0 was used for general data analysis and all statistical analysis. Microscopy images were analyzed with ImageJ.

For manuscripts utilizing custom algorithms or software that are central to the research but not yet described in published literature, software must be made available to editors/reviewers. We strongly encourage code deposition in a community repository (e.g. GitHub). See the Nature Research guidelines for submitting code & software for further information.

Data

Policy information about availability of data

All manuscripts must include a data availability statement. This statement should provide the following information, where applicable:

- Accession codes, unique identifiers, or web links for publicly available datasets
- A list of figures that have associated raw data
- A description of any restrictions on data availability

The genomic data generated in this study is available online under accession code PRJNA513342. RNA-Seq data is available online in the NCBI GEO repository under accession code GSE124869. All strains generated in this study are available from the authors upon request.

Field-specific reporting

Please select the one below that is the best fit for your research. If you are not sure, read the appropriate sections before making your selection.

☒ Life sciences ☐ Behavioural & social sciences ☐ Ecological, evolutionary & environmental sciences

For a reference copy of the document with all sections, see nature.com/documents/nr-reporting-summary-flat.pdf

Life sciences study design

All studies must disclose on these points even when the disclosure is negative.

Sample size	No statistical method was used for the predetermination of sample sizes. Sample sizes varied between experiments and were selected based on previous experience with the model and variability of the infection dynamics and literature reports.
Data exclusions	No data were excluded
Replication	Data from animal infections was pooled and presented in the manuscript. Where murine experiments were repeated, the trend was not different between experiments. In vitro experiments were repeated 3-5 times and results were reliably reproduced.
Randomization	For animal infections, inbred animals were received from Jackson Laboratories and distributed between cages. Cages were randomly assigned to groups.
Blinding	Investigators were not blinded to the study, as experimental parameters were exact measurements of weight, bacterial burden or time to mortality, and not subjective measures of behavior. Euthanasia of animals was performed according to strict guidelines, as outlined in out animal use protocol. The pathology scoring was performed by a trained veterinary pathologist, who was blinded to the study design.

Reporting for specific materials, systems and methods

We require information from authors about some types of materials, experimental systems and methods used in many studies. Here, indicate whether each material, system or method listed is relevant to your study. If you are not sure if a list item applies to your research, read the appropriate section before selecting a response.

Materials & experimental systems

n/a	Involved in the study	
☐	☒	Antibodies
☒	☐	Eukaryotic cell lines
☒	☐	Palaeontology
☐	☒	Animals and other organisms
☐	☒	Human research participants
☒	☐	Clinical data

Methods

n/a	Involved in the study	
☒	☐	ChIP-seq
☒	☐	Flow cytometry
☒	☐	MRI-based neuroimaging

Antibodies

Antibodies used	Rabbit anti Staphylococcus aureus Fibronectin binding protein polyclonal serum. Antibody described in DOI 10.1128/IAI.68.3.1156-1163.2000.<br>Donkey anti Rabbit IRDye800 secondary antibody (Licor BioSciences).
Validation	Antibodies have not been validated.

Animals and other organisms

Policy information about studies involving animals; ARRIVE guidelines recommended for reporting animal research

Laboratory animals	Female BALB/C mice, received from Jackson Laboratories at 8-10 weeks upon arrival.
Wild animals	Study did not involve wild animals
Field-collected samples	Study did not involve samples collected in the field
Ethics oversight	All animal protocols (protocol 2017-028) were reviewed and approved by the University of Western Ontario Animal Use Subcommittee, a subcommittee of the University Council on Animal Care.

Note that full information on the approval of the study protocol must also be provided in the manuscript.

Human research participants

Policy information about studies involving human research participants

Population characteristics	Healthy adult volunteers.
Recruitment	Adult volunteers responded to a poster advertising the study.
Ethics oversight	Human blood was obtained from healthy adult volunteers, with written permission and in compliance with protocol 109059 approved by the Office of Research Ethics at the University of Western Ontario.

Note that full information on the approval of the study protocol must also be provided in the manuscript.
